# Supplementary figures and images for: Differential gene expression analysis by RNA-seq reveals the importance of actin cytoskeletal proteins in erythroleukemia cells
Source: PeerJ. 2017 Jun 27;5:e3432. doi: 10.7717/peerj.3432 (PMC5490462; doi:10.7717/peerj.3432)

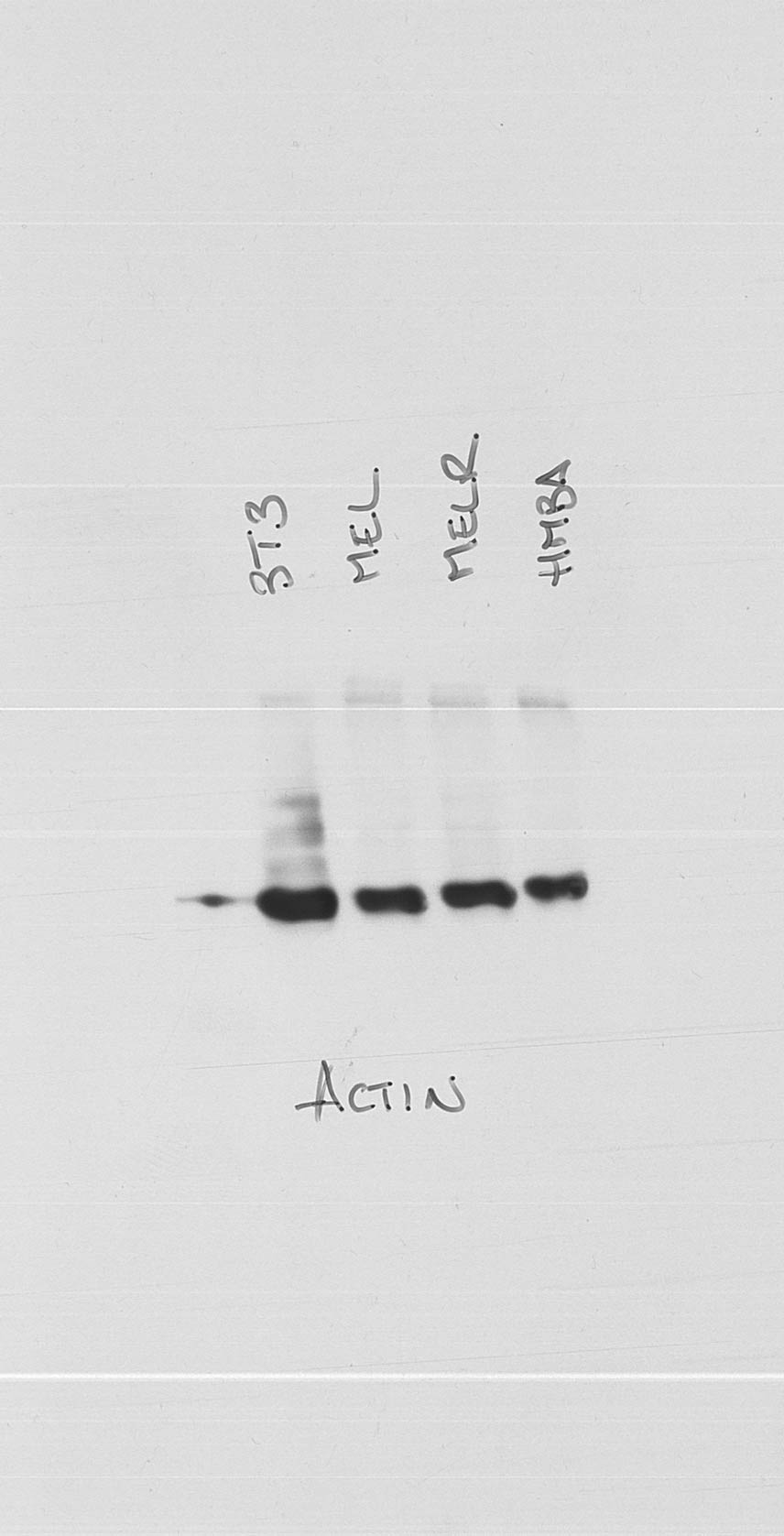

Supplement: Supplemental Information 1 — Raw data for Fig. 8. [file peerj-05-3432-s001.jpg]

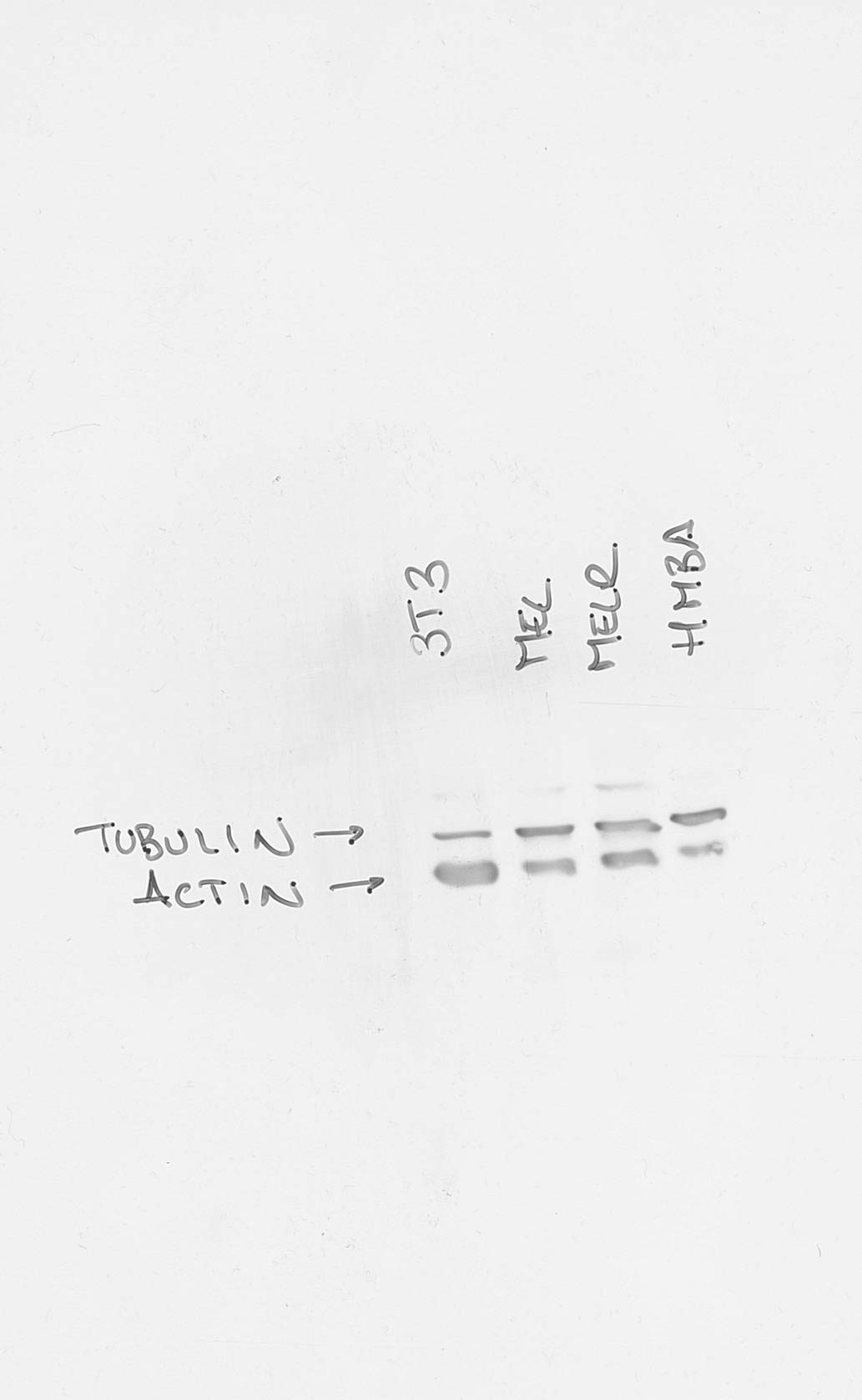

Supplement: Supplemental Information 2 — Raw data for Fig. 8. [file peerj-05-3432-s002.jpg]

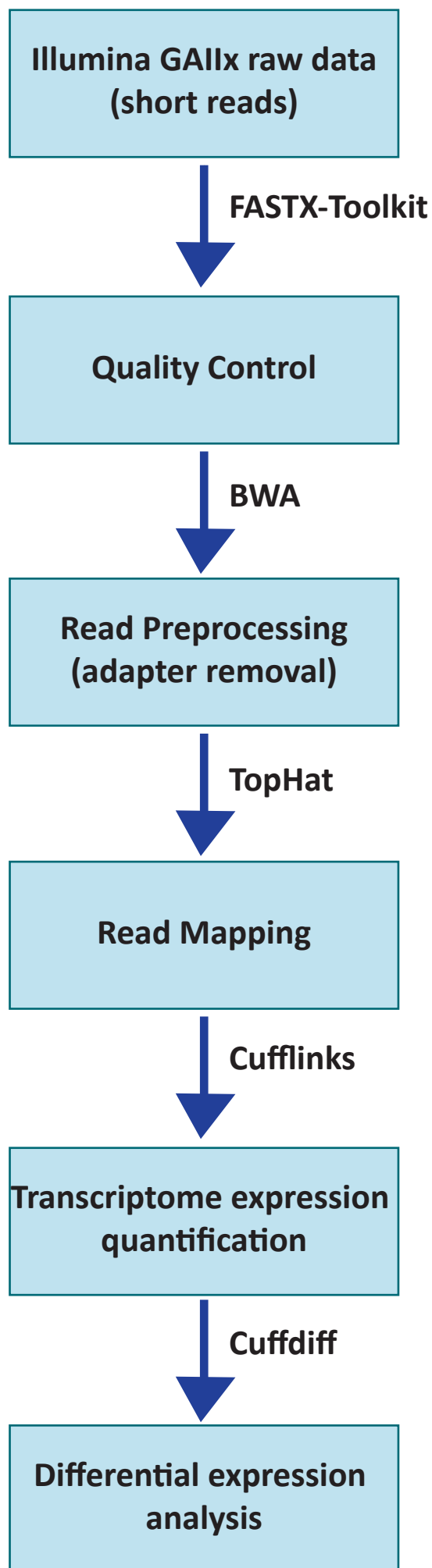

Supplement: Figure S1 — The programs used for each step are detailed to the right of the workflow. [file peerj-05-3432-s003.pdf]

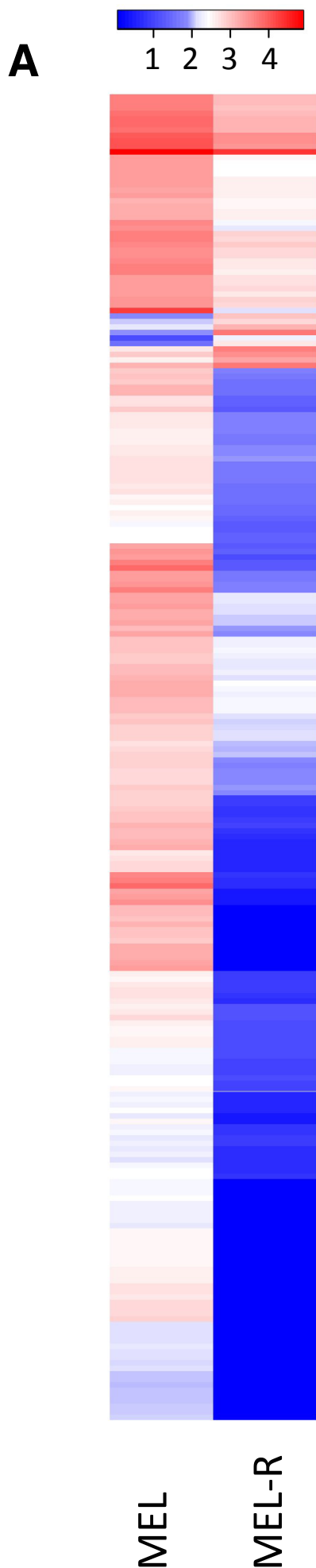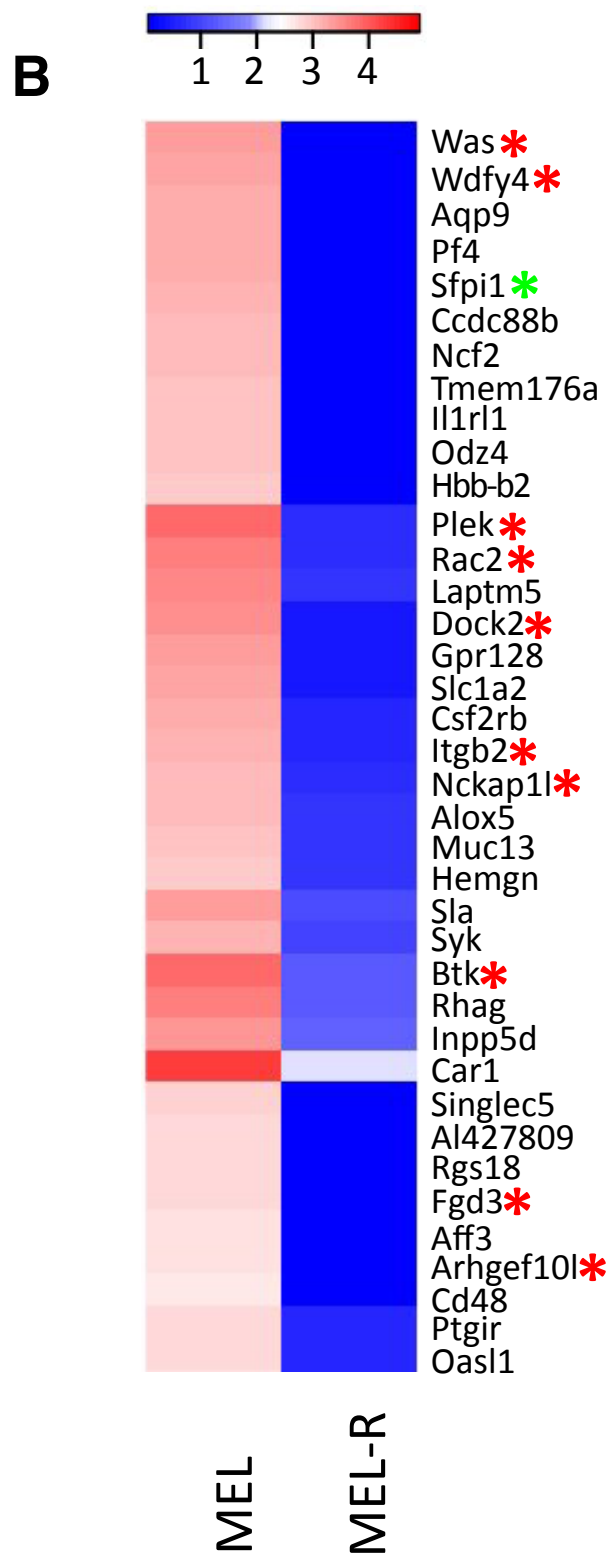

Supplement: Figure S2 — (A) Clusters of differentially expressed genes obtained with DESeq. (B) Heat map zoomed to amplify the genes with higher expression values. Genes related to the actin cytoskeletal network are indicated by red asterisks. Sfpi/PU.1 is marked with a green asterisk. The color scales shown in all maps illustrate the log10 DESeq counts. Red and blue colors represent high and low expression, respectively. [file peerj-05-3432-s004.pdf]

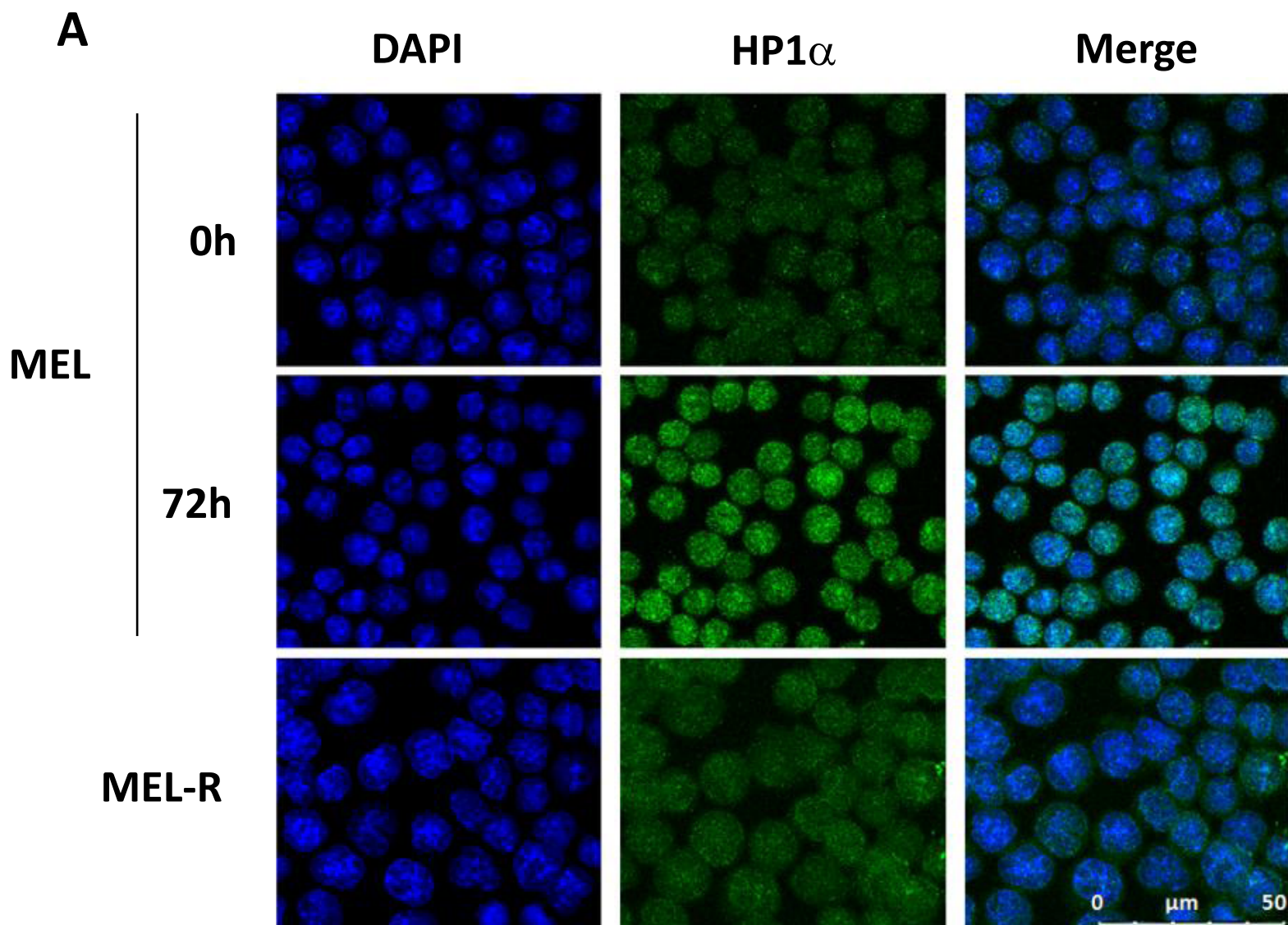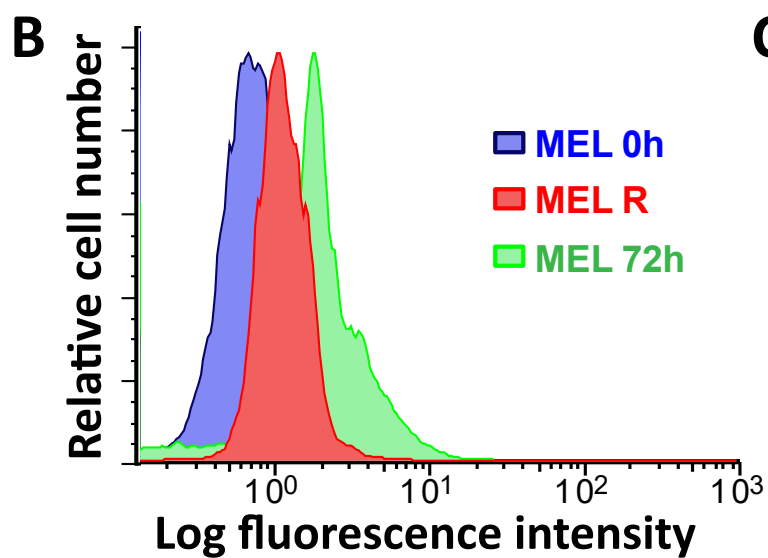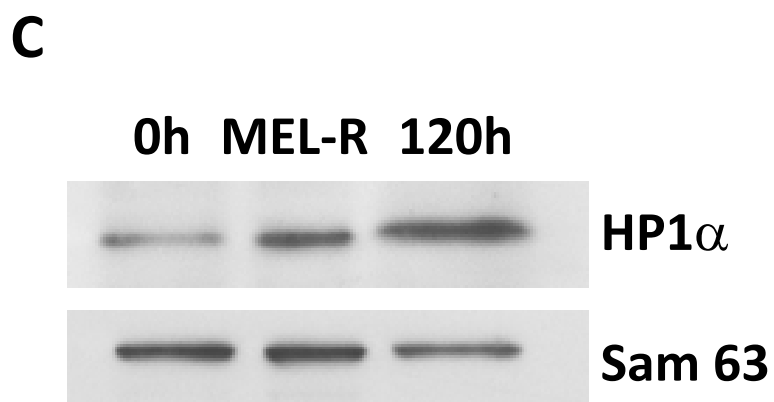

Supplement: Figure S3 — (A) Confocal immunofluorescence microscopy of untreated (0 h) or HMBA-treated MEL (72 h) and MEL-R cells stained with a mouse monoclonal anti-HP1 α antibody (green). Nuclear DNA was stained with DAPI (blue). Scale bar is 50 µm. (B) Flow cytometer analysis of HP1 α fluorescence levels in the samples described in (A). (C) Western blot for HP1 α protein expression in undifferentiated MEL (0 h), MEL differentiated (120 h) and MEL-R cells. Anti-Sam63 was used as a loading control. [file peerj-05-3432-s005.pdf]
