# Supplementary material for: Differential gene expression analysis by RNA-seq reveals the importance of actin cytoskeletal proteins in erythroleukemia cells
Source: PeerJ. 2017 Jun 27;5:e3432. doi: 10.7717/peerj.3432 (PMC5490462; doi:10.7717/peerj.3432)
Supplement: Table S1 [file peerj-05-3432-s006.docx]

| **Gene Symbol** | **Locus** | **Forward**  **(5’-3’)** | **Reverse**  **(5’-3’)** | **Amplicon size (bp)** |
| --- | --- | --- | --- | --- |
| ***Was*** | X:7658591-7667617 | GGATCTGCGGAGCTTGTTCTC | AGACCTCCCTGGTCCTCAAT | 105 |
| ***Plek*** | 11:16871208-16908721 | CTTGAGAGGCTGTGTGGTGA | ATTACTTGCAGGCAGCCACT | 121 |
| ***Arhgef10l*** | 4:140070399-140221820 | TAATGGAGATGGAGCCCAAG | AGCGATCTGGAACATGGAGT | 101 |
| ***Rac2*** | 15:78389598-78403213 | CCTAGGTACGGCACCACT | GGATCTGACCAACCTGGAGC | 98 |
| ***Dock2*** | 11:34126863-34414545 | AAACAAGGGAGCTACGCTGA | ATTCTGGGTCAGCTTTGTGG | 115 |
| ***Btk*** | X:131076879-131117679 | TGGCTGCCTCTTGAACTACC | AAGGAACTGCTTCGACTCCA | 118 |
| ***Nckap1l*** | 15:103284255-103329231 | ACTTTGGCTGGCTACAATGC | AAGCATCTGCACCCAAAAAC | 121 |

**Table S1.** *List of actin cytoskeletal primers used for qRT-PCR*
