# Supplementary material for: Differential gene expression analysis by RNA-seq reveals the importance of actin cytoskeletal proteins in erythroleukemia cells
Source: PeerJ. 2017 Jun 27;5:e3432. doi: 10.7717/peerj.3432 (PMC5490462; doi:10.7717/peerj.3432)
Supplement: Table S2 [file peerj-05-3432-s007.docx]

**Table S2 .** *List of histone primers used for RT-qPCR analysis.*

| **Gene Symbol** | **Locus** | **Forward (5’-3’)** | **Reverse (5’-3’)** | **Amplicon size (bp)** |
| --- | --- | --- | --- | --- |
| ***Hist1h1c*** | 13:23830675-23832236 | TGGAAGTTTGGGAGAAGGTG | TCTCAGTCCCGAGTCCAACT | 119 |
| ***Hist1h1d*** | 13:23646900-23649877 | CGTCTACGTTTCAATCTAGCATTTC | TGAGGCTCGAATACAAGATCC | 66 |
| ***Hist1h2ba*** | 13:24025641-24026025 | GTGGCGTTCACACAACAAG | GCAGCCCTACCGACTGTTAC | 120 |
| ***Hist1h2bc*** | 13:23776067-23784349 | CTCCAAGTGATCCTGCCAAG | CCATTTGCACTGTCTTGAGG | 90 |
| ***Hist1h2bg*** | 13:23663268-23663883 | CGGTACTAAAAGGCCAGACG | TTCCTTATTGGCTGCAGAGG | 105 |
| ***Hist1h2bj*** | 13:22135098-22135479 | CCGACACCGGTATCTCCTC | GTCGAGCGCTTGTTGTAATG | 114 |
| ***Hist1h2bk*** | 13:22127755-22128136 | CGAGGTTTTGGGAGTTGTGT | AGTGAAATGCCCTTGTCTGG | 90 |
| ***Hist1h2bl*** | 13:21807631-21808012 | AATTCGCAGCAGCCTGTAGT | GGTTGGGGTGTGAATACCAG | 90 |
| ***Hist1h2bn*** | 13:21845991-21846372 | CAGCCTGTGCAGACACACTT | CCTTCTTGGACCCCTTCTTC | 120 |
| ***Hist1h2bp*** | 13:21879356-21881082 | TGCCTGAGCCTGTTAAGTCC | CACCGAGTAGCTCTCCTTGC | 119 |
| ***Hist2h2aa1*** | 3:96043638-96044216 | GCCCGCGTCTCTGTGATA | GGCTACCGTGACACAACTCTT | 95 |
| ***Hist2h2aa2*** | 3:96049503-96049896 | GCCCGCGTCTCTGTGATA | GGCTACCGTGACACAACTCTT | 94 |
| ***Hist2h2be*** | 3:96025043-96027661 | CTTCTTGGAGTTCTTTTTAGACTAGGA | AAAGCCTAGAACACGGAAACC | 71 |
| ***Hist2h3c2*** | 3:96042038-96043050 | CATTCCCCACAAAGGCTCT | GCTCTAAGCGAAGTAAACAGCTC | 70 |
| ***Hist3h2a*** | 11:58768186-58770176 | TGGAGGGAGGTGTACTAGGG | TTGGGTAGTGGTTGTGCATTT | 71 |
| ***H1f0*** | 15:78858641-78860934 | CAAAGCCCCAAGCAAGAA | CAACCTTGGGCTTTTTGG | 93 |
| ***H2afx*** | 9:44142797-44144160 | GTTGCTGGCCTCATACCAGT | GCCGGGAGGTATTCCTAGAG | 111 |
| ***H3f3b*** | 11:115880075-115885818 | CAGGATTTCAAAACCGACTTG | CCCACCAGGTATGCTTCG | 108 |
