# Supplementary material for: Differential gene expression analysis by RNA-seq reveals the importance of actin cytoskeletal proteins in erythroleukemia cells
Source: PeerJ. 2017 Jun 27;5:e3432. doi: 10.7717/peerj.3432 (PMC5490462; doi:10.7717/peerj.3432)
Supplement: Table S3 [file peerj-05-3432-s008.docx]

**Table S3.** *List of Dnmts and Tets primers used for qRT-PCR*

| **Gene Symbol** | **Locus** | **Forward**  **(5’-3’)** | **Reverse**  **(5’-3’)** | **Amplicon size (bp)** |
| --- | --- | --- | --- | --- |
| ***Dnmt1*** | 9:20907206-20952979 | AAAGTGTGATCCCGAAGATCAAC | TGGTACTTCAGGTTAGGGTCGTCTA | 79 |
| ***Dnmt3a*** | 12: 3891744-3914443 | TGCTACATGTGCGGGCATAA | GGAGTCGAGAAGGCCAGTCTT | 70 |
| ***Dnmt3b*** | 2:153649449-153687730 | CCCAAGTTGTACCCAGCAATTC | TGCAATTCCATCAAACAGAGACA | 75 |
| ***Tet1*** | 10:62804570-62880014 | GCGTGAAGCTCAAACATCAA | GTGCAGCTTCCTGTTCCTTC | 143 |
| ***Tet2*** | 3:133463677-133544390 | GATCCGTCATCTGAGGCTCA | TGGCCTGTTGACTCAAATGC | 103 |
| ***Tet3*** | 6:83362374-83441678 | GCAAGGAGGGGAAGAGTTCT | ATCACGGCGTTCTGACAATG | 127 |
