# Supplementary material for: Differential gene expression analysis by RNA-seq reveals the importance of actin cytoskeletal proteins in erythroleukemia cells
Source: PeerJ. 2017 Jun 27;5:e3432. doi: 10.7717/peerj.3432 (PMC5490462; doi:10.7717/peerj.3432)
Supplement: Table S3 [file peerj-05-3432-s009.docx]

| **Gene Symbol** | **Region** | **Forward**  **(5’-3’)** | **Reverse**  **(5’-3’)** | **Amplicon size (bp)** |
| --- | --- | --- | --- | --- |
| ***Btk*** | 1 | AAAAATGTTTTTTTAGAAAATGATTTAGTA | ACCCTCTCTAAAACAAAAAAATAAC | 240 |
|  | 2 | TGTAAAGAGGTTTTAGGTAAATGT | TTAATACAAATAACTCCCCAAAC | 300 |
| ***Plek*** | 1 | TTTAGGATTTGTGGGGATAATAAAG | CAACCCATACTTTCCTAATAATAAAAATAA | 298 |
|  | 2 | AATTTTTAAGTAGGATAGTTTTAGG | CCTTTTTACAAAAAAATCTCTTTCAC | 260 |
|  | 3 | TTTAGAGAGGGGAAAAGGATGTTAT | ACAACCCACATAAATTTCCAAATAT | 240 |
| ***Was*** | 1 | AATGTAATGGTTTTTTAGTATTTGGAGTT | TTCCTTCTAACAAACTTAAATCAACCT | 196 |
|  | 2 | TAAGTTTGTTAGAAGGAAATGGTTT | ACTACCTTCTACCTTAATCCTCTCCA | 282 |
|  | 3 | TTTTGAAGGAGAAGTTTTGTAGATG | TCTAAAACAAACATAAACCCTCCTC | 203 |

**Table S4.** *List of primers used for bisulfite analysis*
